# Genomic Sequencing and Phylogenomics of Cowpox Virus

**DOI:** 10.3390/v14102134

**Published:** 2022-09-28

**Authors:** Diana Diaz-Cánova, Carla Mavian, Annika Brinkmann, Andreas Nitsche, Ugo Moens, Malachy Ifeanyi Okeke

**Affiliations:** 1 Molecular Inflammation Research Group, Department of Medical Biology, UiT—The Arctic University of Norway, N-9037 Tromsø, Norway; 2 Emerging Pathogens Institute, Department of Pathology, College of Medicine, University of Florida, Gainesville, FL 32610, USA; 3 Highly Pathogenic Viruses, Centre for Biological Threats and Special Pathogens, WHO Reference Laboratory for SARS-CoV-2 and WHO Collaborating Centre for Emerging Infections and Biological Threats, Robert Koch Institute, 1335 Berlin, Germany; 4 Section of Biomedical Sciences, Department of Natural and Environmental Sciences, School of Arts and Sciences, American University of Nigeria, Yola PMB 2250, Nigeria

**Keywords:** phylogenetic, orthopoxvirus, poxviridae, molecular clock, Fennoscandian, phylodynamics, cowpox virus

## Abstract

*Cowpox virus* (CPXV; genus *Orthopoxvirus*; family *Poxviridae*) is the causative agent of cowpox, a self-limiting zoonotic infection. CPXV is endemic in Eurasia, and human CPXV infections are associated with exposure to infected animals. In the Fennoscandian region, five CPXVs isolated from cats and humans were collected and used in this study. We report the complete sequence of their genomes, which ranged in size from 220–222 kbp, containing between 215 and 219 open reading frames. The phylogenetic analysis of 87 orthopoxvirus strains, including the Fennoscandian CPXV isolates, confirmed the division of CPXV strains into at least five distinct major clusters (CPXV-like 1, CPXV-like 2, VACV-like, VARV-like and ECTV-Abatino-like) and can be further divided into eighteen sub-species based on the genetic and patristic distances. Bayesian time-scaled evolutionary history of CPXV was reconstructed employing concatenated 62 non-recombinant conserved genes of 55 CPXV. The CPXV evolution rate was calculated to be 1.65 × 10^−5^ substitution/site/year. Our findings confirmed that CPXV is not a single species but a polyphyletic assemblage of several species and thus, a reclassification is warranted.

## 1. Introduction

*Cowpox virus* (CPXV) is an orthopoxvirus species, belonging to the subfamily *Chordopoxvirinae* of the family *Poxviridae* [1]. Orthopoxvirus (OPXV) comprises several species from the New World and Old World. The most representative species from the New World are raccoonpox virus (RCNV), volepox virus (VPXV) and skunkpox virus (SKPV) [2]. Within Old World OPXV, there are several species: ectromelia virus (ECTV), vaccinia virus (VACV), monkeypox virus (MPXV), variola virus (VARV), taterapox virus (TATV), camelpox virus (CMLV) and CPXV [3,4,5]. In the last decade, new OPXV species were discovered in the United States (alaskapox virus, AKPV), Italy (abatino macacapox virus, Abatino) and Georgia (akhmeta virus, AKMV) [6,7,8].

The most notable member of OPXV genus is VARV, the etiologic agent of smallpox. However, after a large, massive vaccination campaign, smallpox was eradicated in 1980 [9]. The last natural cases of smallpox in humans were in Somalia in 1977 [10]. OPXV species, such as CPXV and MPXV, can cause zoonotic diseases [11,12,13,14]. MPXV and CPXV are the causative agents of monkeypox and cowpox, respectively, and have a wide host range [13,15]. Recently, a multi-country human monkeypox outbreak in 50 countries has been reported [16]. Compared to MPXV that occurs in Central and Western Africa [17], CPXV is endemic of Eurasia, mainly present in Europe [12,18,19,20,21,22,23]. The natural reservoirs of CPXV are wild rodents [18,24]. Nevertheless, CPXV is also able to infect felines, monkeys, dogs, alpacas, rats, horses and humans [12,25,26,27,28,29]. The first zoonotic case was reported in the Netherlands in 1985, where CPXV was transmitted from a domestic cat to a woman [30]. In Fennoscandian, human cases of CPXV infections have been reported (CPXV-No-H1, CPXV-No-H2, CPXV-Swe-H1 and CPXV-Swe-H2) as well as feline cases (CPXV-No-F1 and CPXV-No-F2) [27,31,32,33,34,35]. CPXV has been classified as a single species; however, it has been proposed that CPXV should be considered as a polyphyletic species [33,35,36,37,38,39,40]. Based on phylogenetic studies, CPXV was divided into at least five clades: CPXV-like 1, CPXV-like 2, ECTV-Abatino-like, VACV-like and VARV-like [38,40,41,42]. Among OPXV, CPXV has the largest genome [43] and contains the highest number of orthopoxviral genes [42,44]. It was suggested that CPXV-like virus was the ancestor of Old World OPXV, except for AKPV and AKMV [39,44,45]. Until now, the evolutionary history of CPXV is still unclear. Most studies have focused on the molecular evolution of VARV, but few studies were focused on OPXV and, specially, on CPXV [39,45,46,47,48,49].

In this study, we present the whole genome sequence of five Fennoscandian CPXV isolates. We determined the phylogenetic relationship of CPXV, including the Fennoscandian isolates, with other OPXV and studied the evolutionary history of CPXV based on the concatenated 62 non-recombinant conserved genes of several representatives CPXV isolates from the different CPXV clades. Furthermore, we propose a new classification of CPXV.

## 2. Materials and Methods

### 2.1. Cell, Virus Culture and DNA Isolation

Five Fennoscandian isolates were used in this study: CPXV-No-H1, CPXV-No-F1, CPXV-No-F2, CPXV-Swe-H1 and CPXV-Swe-H2. The isolates were cultured on a monolayer of Vero cells (ATCC No. CCL-81), and the viral DNA was extracted from semi-purified virions, as previously described [34,40]. The origin of the five CPXV isolates has been described elsewhere [27,31,32,33,34].

### 2.2. Whole Genome Sequencing, Genome Assembly and Genome Annotation

The genomes of the five Fennoscandian isolates (CPXV-No-H1, CPXV-No-F1, CPXV-No-F2, CPXV-Swe-H1 and CPXV-Swe-H2) were sequenced using Oxford Nanopore Technology GridION (ONT; Oxford, UK) and Illumina MiSeq using reagent kit v3 with 2 × 300 bp paired-end reads, as previously described [40]. Illumina sequencing was performed at the Norwegian Sequencing Centre, Oslo, and Nanopore sequencing was performed at the Genomics Support Centre Tromsø at UiT—The Arctic University of Norway. The genomes were assembled using SPAdes v3.15.3 [50] and annotated with Genome Annotation Transfer Utility (GATU) [51], as previously reported [40].

### 2.3. Gene Content Comparison

The five Fennoscandian CPXV genomes were compared to CPXV-Br genome. Predicted CDS from five CPXV isolates were extracted, translated into amino acid sequences and compared to the CPXV-Br proteins using BLASTp (ncbi-blast+ v2.11.0) [52].

### 2.4. Phylogenetic Analysis, Patristic and Genetic Distances

A total of 87 OPXV genomes, including the five Fennoscandian genomes, were used in this study (Table S1). Eighty-two OPXV genomes were retrieved from the Viral Orthologous Clusters (VOCs) database [53], with the exception of CPXV_GerMygEK938_17 (retrieved from GenBank). The genes and genomes were aligned using MAFFT v7.450 (with default parameters) [54], as implemented in Geneious Prime 2022.0.2. Four different alignments were used to build the phylogenetic trees: (1) 87 OPXV whole genome alignment, (2) 87 OPXV core genome alignment, (3) OPXV orthologous gene alignment (Table S2) and (4) 62 conserved genes alignment (Table S3), as previously described [40].

Recombination detection program 4 (RDP4) [55] was used to detect genome-wide recombination in the datasets. Recombination events identified by 5 of 7 methods (RDP [56], GENECONV [57], Bootscan [58], MaxChi [59], Chimaera [60], SiScan [61] and 3Seq [62]) with significant *p*-values (*p* ≤ 0.01) were considered credible evidences of recombination. Whole genome, core genome and orthologous gene alignments were generated without removing the putative recombinant regions.

The conserved gene alignment was generated by examining the 90 Chordopoxvirus (ChPV) conserved genes for recombination using RDP4 [55], as described above. The 62 conserved genes identified as non-recombinant were aligned singly and the 62 single gene alignments were concatenated to generate the conserved gene dataset.

Gblocks 0.91b was used to remove poorly aligned positions from 87 OPXV whole and core genome alignments [63]. The presence of phylogenetic signal of the datasets was assessed by likelihood mapping analysis with the evaluation of 2000 random quartets using IQ-TREE v.2.0.3. [64] (Figure S1). The best-fit nucleotide substitution model for the alignment data was selected using the modelTest-NG v.0.1.6 [65]. Two inference methods, maximum likelihood (ML) and Bayesian inference (BI), were conducted with RAxML v8.2.12 [66] using a rapid bootstrap algorithm [67] and MrBayes v3.2.7 [68], respectively, as previously described [40]. The Markov Chain Monte Carlo (MCMC) analysis was run until reaching convergence. The phylogenetic trees were visualized applying FigTree v1.4.4 (http://tree.bio.ed.ac.uk/software/figtree/, accessed on 19 February 2021). The BI phylogenetic tree based on the OPXV orthologous genes was not built because MCMC analysis did not reach convergence after 50,000,000 generations.

Patristic distances between different groups were calculated from the ML/BI trees of concatenated 62 conserved non-recombinant genes using the program Patristic version 1.0 [69]. The genetic distances between the different groups were estimated by p-distances, as implemented in MEGA version 11 [70]. For patristic and genetic distances, the distances were averaged across taxa to produce a single value. The genetic and patristic distances between TATV and CMLV were used as threshold values since they are closest and distinct OPXV species. These threshold values were used to compare the distance between CPXV clusters and OPXV species and separate them in different sub-species if they were equal or greater than TATV-CMLV threshold values.

### 2.5. Phylodynamic Evolutionary Analysis of CPXV

A Bayesian MCMC inference method implemented in BEAST 1.10.4 [71] was used to estimate evolutionary rates and the divergence times. Evolutionary analysis was carried out on alignment of concatenated 62 conserved non-recombinant genes of 55 CPXV strains (listed in Table S4). The temporal signal was assessed from the ML tree of 62 conserved genes of 55 CPXV by regression of genetic divergence (root-to-tip genetic distance) and the sampling date using TempEst v.1.5.3 [72] (Figure S2). In the analysis, we did not include other OPXV species because the dataset did not contain temporal signal (correlation coefficient = −0.15, value of R2 = 0.02). The presence of phylogenetic signal of the dataset was evaluated using IQ-TREE v.2.0.3. [64], as described above (Figure S2).

The Bayesian phylodynamic analysis was calibrated using the following parameters: log-normal relaxed clock, coalescent Bayesian skyline population, HKY substitution model and four gamma categories. MCMC chain was run for 1 billion generations. The effective sampling size (ESS) values were checked in Tracer v1.7.2 [73]. Only the Effective Sampling Size (ESS) values > 200 (after burn-in) were accepted. The maximum clade credibility (MCC) tree was generated using TreeAnnotator v1.10.4, visualized using FigTree v1.4.4 and edited graphically using the ggtree package available in R [74].

## 3. Results

### 3.1. Genome Assembly, Genome Annotation and Gene Content

The whole genomes of five Fennoscandian CPXV isolates (CPXV-No-H1, CPXV-No-F1, CPXV-No-F2, CPXV-Swe-H1 and CPXV-Swe-H2) were assembled, and the coverage of the assembled genomes varied from 300X to 2400X, as shown in Table 1. The genome size of the Fennoscandian CPXV isolates ranges from 220,808 to 222,178 bp and the length of inverted terminal repeats (ITRs) were approximately 8 kbp (Table 1). The whole genome sequences of these isolates are available in GenBank, with Accession Number: OP125537, OP125538, OP125539, OP125540, OP125541.

Gene annotation of the five Fennoscandian CPXV genomes (CPXV-No-H1, CPXV-No-F1, CPXV-No-F2, CPXV-Swe-H1 and CPXV-Swe-H2) revealed 212, 219, 217, 217 and 217 predicted coding sequences (CDS), respectively. A comparison of the predicted CDS of the five Fennoscandian CPXV isolates with the CPXV-Br genome is shown in Table S5. The genome content of the five Fennoscandian CXPV isolates was similar to CXPV-Br genome. The majority of predicted CDS of the five CXPV strains were found to have homologs in CPXV-Br, except for few predicted CDS. *NoF1-009, NoF2-009* and *NoH1-008*, present in the Norwegian isolates, were homologs of *EVM004* that encodes a BTB Kelch-domain containing protein. The Swedish isolates contain a CDS (*SweH1-210* and *SweH2-210*) that was homolog of *CPXV-GRI-K3R* (codes for CrmE protein). The five Fennoscandian isolates contain a homolog of *VACV-Cop O3L*, encoding a virus entry/fusion complex component.

The five Fennoscandian CPXV strains lacked homologs of *CPXV001* and *CPXV216*. Furthermore, *CPXV002* and *CPXV191* (*CrmC*) were absent in CPXV-No-H1 and CPXV-No-F1 genomes, respectively.

### 3.2. Phylogenetic Analysis

**Figure 1:**
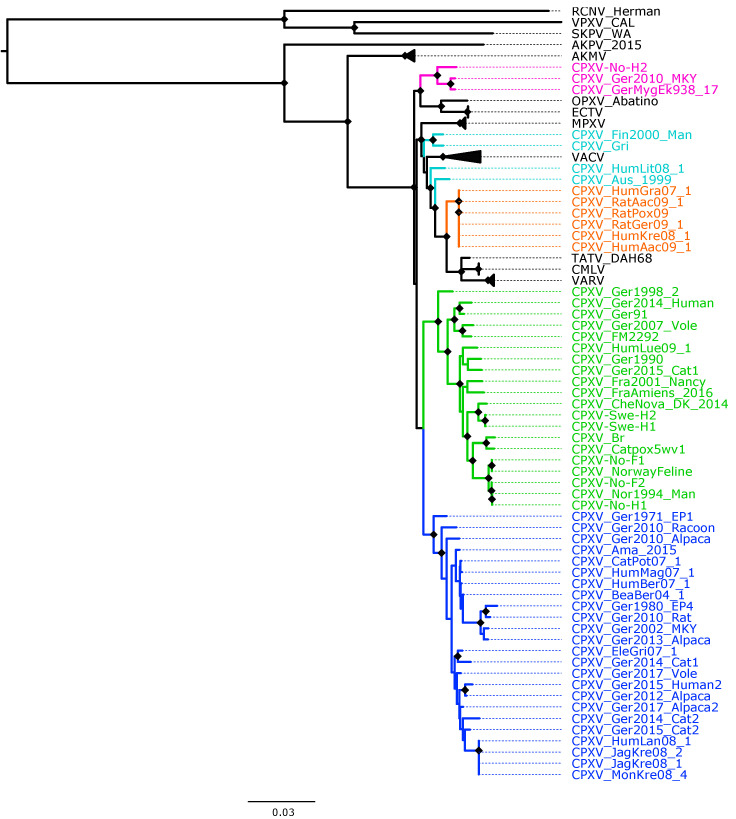
Maximum likelihood phylogenetic tree of 62 conserved genes from 87 orthopoxviruses. Bootstrap values were inferred from 1000 rapid bootstrap replicates. Diamonds at the nodes indicate bootstrap values > 80%. The scale indicates substitution per site. The main five cowpox virus (CPXV) clusters were highlighted in different colors: pink (Ectromelia-Abatino-like CPXV), blue (CPXV-like 1), green (CPXV-like 2), turquoise blue (Vaccinia-like CPXV) and orange (Variola-like CPXV).

The recombination analysis evidenced the extensive recombination in OPXV core genomes (Figure S3) as well as in the datasets of OPXV whole genomes and orthologous genes (data not shown). Recombination regions were not removed from the alignments used to generate the phylogenetic trees for the whole genome, core genome and orthologous genes. To examine if the recombinant regions in the three datasets biased the phylogenetic signal, we generated some fourth data, in which 62 OPXV conserved genes without any evidence of recombination were used in phylogenetic reconstruction, as described in methods. The ML and BI phylogenetic trees built from concatenated 62 conserved genes without recombination is shown in Figure 1 and Figure S4, respectively.

**Figure 2:**
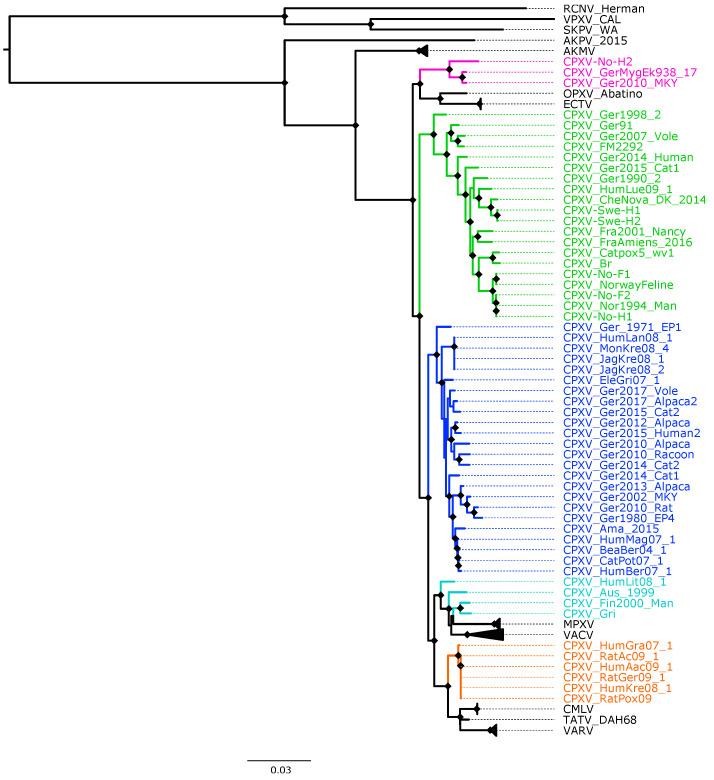
Maximum likelihood phylogenetic tree of 87 orthopoxvirus core genome. Bootstrap values were inferred from 1000 rapid bootstrap replicates. Diamonds at the nodes indicate bootstrap values > 80%. The scale indicates substitution per site. The main five cowpox virus (CPXV) clusters were highlighted in different colors: pink (Ectromelia-Abatino-like CPXV), blue (CPXV-like 1), green (CPXV-like 2), turquoise blue (Vaccinia-like CPXV) and orange (Variola-like CPXV).

The topology of the phylogenetic trees based on 87 OPXV core genomes (Figure 2 and Figure S5) was identical to that of trees generated from 87 OPXV whole genomes (Figures S6 and S7) and similar to that of the phylogenetic tree built based on OPXV orthologous genes (Figure S8). Whereas the topology of phylogenetic trees based on 62 conserved genes (Figure 1 and Figure S4) slightly differed from that of the phylogenetic trees generated from 87 OPXV core genomes (Figure 2 and Figure S5).

As expected, in all phylogenetic trees, the New World and Old World OPXV were separated and AKPV and AKMV clades were placed between them (Figure 1, Figure 2 and Figures S4–S8). Within the Old OPXV, the strains from the same species formed distinct clades, except for CPXV strains. They formed separated clusters with different OPXV species such as VACV, VARV, ECTV and Abatino. CPXV isolates were separated in five clusters: ECTV-Abatino-like CPXV, CPXV-like 1, CPXV-like 2, VACV-like CPXV and VARV-like CPXV. Even though CPXV strains from VACV-like did not form a cluster, they were closely related to VACV clade. ECTV/Abatino group was clustered with ECTV-Abatino-like clade, which includes one Fennoscandian isolate (CXPV-No-H2) and two German isolates. ECTV/Abatino/ECTV-Abatino-like CPXV clade clustered with a major clade that contains: CPXV-like 2, CPXV-like 1, VACV-like, VACV, MPXV, VARV-like, VARV, TATV and CMLV clusters (PP = 1.0 and bootstrap values = 100%). In the phylogenetic trees based on 87 OPXV core genomes (Figure 2 and Figure S5), CPXV-like 2 was separated from the other CPXV clusters and OPXV. Furthermore, CPXV-like 1 clade was sister to a major clade that comprised VACV-like, VACV, MPXV, VARV-like, TATV, CMLV and VARV (PP = 1.0 and bootstrap values = 100%). Within this major clade VACV-like/VACV/MPXV cluster was separated from VARV-like, TATV, CMLV and VARV. Whereas the phylogenetic trees generated from 62 conserved genes (Figure 2 and Figure S6) showed that CPXV-like 1 and CPXV-like 2 were sister clades (PP = 1 and bootstrap values = 70%) and these clustered together with a clade that contains VACV-like, VACV, MPXV, VARV-like, VARV, TATV and CMLV, but with low bootstrap support (48%) and PP of 0.93. In comparison to a phylogenetic tree built from 87 OPXV core genomes, VACV-like/VACV/MPXV did not form separate from VARV-like.

All Fennoscandian CPXV isolates except CPXV-No-H2 were grouped into CPXV-like 2 clade (Figure 1, Figure 2 and Figures S4–S8). This clade also contains CPXV strains from Germany, Denmark, Russia, The United Kingdom (UK) and France. Within CPXV-like 2, CPXV-Ger1998_2 formed a deeper single branch and the remaining CPXV isolates were divided in two main sub-clusters. In the phylogenetic trees built from 87 OPXV core genomes (Figure 2 and Figure S5), the sub-cluster one contained three German isolates (CPXV_Ger91, CPXV_Ger2007_Vole and CPXV_FM2292) and sub-cluster two comprised 16 CPXV isolates, including the five Fennoscandian CPXV isolates reported in this study (CPXV_Ger2014_Human, CPXV_Ger2015_cat1, CPXV_Ger1990_2, CPXV_HumLue09_1, CPXV_CheNova_DK_2014, CPXV-Swe-H1, CPXV-Swe-H2, CPXV-Fra2001-Nancy, CPXV-FraAmiens_2016, CPXV-Catpox5-wv1, CPXV-Br, CPXV-No-F1, CPXV-Norwayfeline, CPXV-No-F2, CPXV-No-H1 and CPXV-Nor1994_Man). Whereas sub-cluster one of the phylogenetic tree based on 62 conserved genes contained an additional CPXV strain, CPXV_Ger0214_Human (Figure 1 and Figure S4). In all phylogenetic trees, the Norwegian isolates were closely related to the UK isolates (CPXV-Br and CPXV- Catpox5_wv1), while Swedish isolates were closer to the Danish isolate. CPXV-like 1 clade was the largest CPXV clade and comprises only German CPXV isolates as well as VARV-like clade. This clade was sister group of VARV/CMLV/TATV. VACV-like contains CPXV strains from Austria, Russia, Finland and Lithuania. These strains were closely related to VACV and MPXV. Compared to VACV-like, VARV-like and ECTV-Abatino-like, CPXV-like 1 and CPXV-like 2 did not cluster together with other OPXV species (Figure 1, Figure 2 and Figures S4–S8). Overall, all phylogenetic trees (based on 87 OPXV whole genomes, core genomes, orthologous genes and conserved genes) showed the five major CPXV clusters and the clustering of the CPXV-like 2 strains were similar. Thus, although recombination among CPXV is extensive, tree topology from datasets with recombinant regions and datasets without evidence of recombination were very similar.

### 3.3. Patristic and Genetic Distances

**Figure 3:**
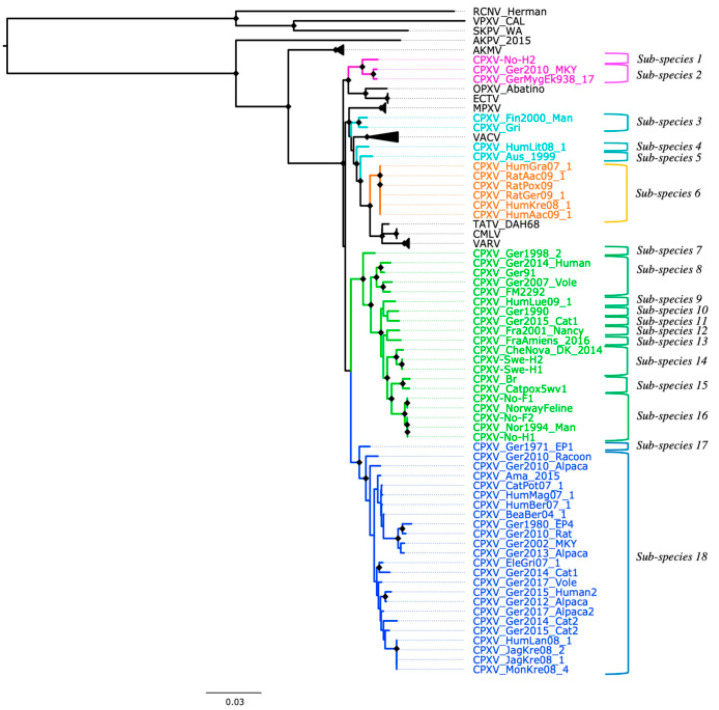
New classification of cowpox virus (CPXV) based on phylogenetic tree inference from 62 conserved genes without evidence of recombination, patristic and genetic distances. Diamonds at the nodes indicate bootstrap values > 80%. The main five CPXV clusters were highlighted in different colors: pink (Ectromelia-Abatino-like CPXV), blue (CPXV-like 1), green (CPXV-like 2), turquoise blue (Vaccinia-like CPXV) and orange (Variola-like CPXV).

Based on the genetic and patristic distances, CPXV strains can be classified into 18 sub-species (Figure 3). The genetic and patristic distances between CPXV clusters and OPXV species were higher than the TATV-CMLV genetic and patristic distance threshold (Tables S6 and S7). Furthermore, the genetic and patristic distances within some CPXV clusters, such as CPXV-like 2, were higher than the threshold values (Table S8). According to the genetic and patristic distances between CPXV-like 2 strains, CPXV-like 2 was further divided into ten sub-species: group one (CPXV-Ger1998_2), group two (CPXV_Ger2014_Human, CPXV_Ger91, CPXV_Ger2007_Vole and CPXV_FM2292), group three (CPXV_HumLue09_1), group four (CPXV_Ger1990_2), group five (CPXV_Ger2015_cat1), group six (CPXV-Fra2001-Nancy), group seven (CPXV-FraAmiens_2016), group eight (CPXV_CheNova_DK_2014, CPXV-Swe-H1 and CPXV-Swe-H2), group nine (CPXV-Catpox5-wv1 and CPXV-Br) and group ten (CPXV-No-F1, CPXV-Norwayfeline, CPXV-No-F2, CPXV-No-H1 and CPXV-Nor1994_Man) (Tables S9 and S10). The isolates were grouped together according to their origin, except for the German and French isolates that were separated into five and two distinct sub-species, respectively.

Similarly, in the ECTV-Abatino-like clade, the genetic and patristic distances between the Norwegian human isolate, CPXV-No-H2, and the German isolates (CPXV_GerMygEk938_17 and CPXV_Ger201_MKY) exceeded the distances between TATV and CMLV (Table S11). Within VACV-like, the distances between the CPXV strains were higher than the threshold values, except for the distances between CXPV-Gri and CPXV-Fin2000-Man (Tables S12 and S13). VACV-like strains were divided into three different sub-species: sub-species one, CPXV_HumLit08_1; sub-species two, CPXV_Aus_1999; sub-species three, CPXV_Gri and CPXV_Fin2000_Man (Figure 3). In CPXV-like 1, CPXV_Ger_1971_EP1 was classified as one sub-species and the remaining CPXV-like 1 strains as another sub-species, according to the genetic and patristic distances (Table S14). However, the patristic distances between CPXV_Ger2010_Alpaca and other CPXV-like 1 strains were higher than the threshold value, but some genetic distances were lower than the threshold values (Tables S15 and S16). VARV-like strains remained together as one sub-species based on the genetic and patristic distances and phylogeny. Curiously, these strains contain a genomic region of approximately 5860 bp that was also identified in some CPXV-like 2 strains (CPXV_Ger91, CPXV_2007_vole, CPXV_FM2291 and CPXV_Fra2001_Nancy), VARV and CMLV.

### 3.4. Evolutionary Analysis of CPXV

The phylodynamic analysis was performed based on the 62 conserved genes of CPXV genomes. The dataset exhibited a positive correlation between the genetic divergence and the sampling time, which indicates the presence of temporal signal in the sequence dataset (correlation efficient = 0.48; R2 = 0.23). The mean evolution rate of CPXV was estimated to be 1.65 × 10^−5^ substitutions per site per year (subs/site/year), with 95% high posterior density interval (HPD) of 4.36 × 10^−7^ − 4.32 × 10^−5^ subs/site/year.

The MCC tree showed that CPXV strains were divided into two main clusters (Figure S9). The minor cluster contained CPXV-like 2 clade (PP = 0.88) and the major cluster comprised ECTV-Abatino-like, VACV-like, VARV-like and CXPV-like 1 clades (PP = 0.89) (Figure S9). However, the emergence date of CPXV as well as major CPXV clusters could not be accurately estimated since the 95% HPD intervals were wide, especially in the deepest nodes. As compared to the 95% HPD intervals tMRCA for recent nodes, tMRCA for deeper internal nodes were quite broad and showed some degree of overlap.

## 4. Discussion

CPXV strains examined in this study were isolated from different countries in Eurasia, with most of CPXV isolates from Germany. We included five CPXV isolates collected from Fennoscandian as well as our previously published CPXV isolate, CPXV-No-H2 [40]. These five Fennoscandian isolates were previously classified as CPXV based on Hind III restriction map of virus DNA, phylogenetic analysis of multiple conserved genes and the possession of two copies of the intact *cytokine response modifier B (CrmB)* gene [33,34,35].

CPXV is classified as one species, but this has been debated in many studies due to its genetic heterogeneity and polyphyletic character [33,35,36,37,38,39,40]. The genetic heterogeneity among CPXV strains could be due to recombination processes [34,35,41] since it is part of the evolution of OPXV [8,34,40,41,43,75,76,77,78,79]. It has been suggested that recombination can affect the accuracy of the phylogenetic inferences [80]. Since the extensive recombination in OPXV genomes has been reported by others [41], we included in our study a dataset of 62 non-recombinant conserved genes to avoid inaccuracy of phylogenetic estimation due the presence of recombination in 87 OPXV whole genomes, core genomes and orthologous genes.

Our phylogenetic analysis using different datasets always showed that CPXV isolates were divided into at least five clusters: CPXV-like 1, CPXV-like 2, VACV-like CPXV, VARV-like CPXV and ECTV-Abatino-like CPXV (Figure 1, Figure 2 and Figures S3–S7). Similar phylogenetic clustering of CPXV has been reported in other studies [40,81]. Three of the five CPXV clusters were closely related to other OPXV species, such as ECTV, Abatino, VARV and VACV. Previous studies have also showed this phylogenetic relationship of CPXV with other OPXV [35,37,38,40,41,43,81].

The German isolates were present in all CPXV clusters, except for VACV-like, while the Fennoscandian CPXV isolates clustered into CPXV-like 2 and grouped into separate clusters according to their country of origin (Norway, Sweden and Denmark), except for CPXV-No-H2. These results are in agreement with the phylogenetic analysis based on single genes (*atip, p4c,* *CrmB, HA,* complete *CHOhr* or partial *CHOhr*) reported in our previous studies [33,35]. However, not all Fennoscandian isolates were closely related. The Norwegian isolates were closely related to the UK strains, whereas the Swedish CPXV isolates were closer to the Danish CPXV isolate. The phylogenetic relationship of the Norwegian and UK isolates has been previously reported [38,41]. In our previous studies the relationship of the Fennoscandian isolates with other CPXV isolates varied depending on the single gene used in the phylogenetic analysis [33,35]. However, in the present study, the phylogenetic relationship between the Norwegian and UK isolates as well as the Swedish and Danish isolates were consistent, regardless of the alignment used (87 OPXV whole genomes, core genomes, orthologous genes or 62 conserved genes).

Genetic and patristic distances have been previously used to examine the diversity of CPXV [35,36,38]. We used the genetic and patristic distances between TATV and CMLV to classify OPXV into the same or different species because they are the closest and recognized OPXV species. Our examination of the genetic and patristic distances between and within CPXV clusters revealed that the five CPXV clusters can be considered distinct CPXV sub-species and that even the CPXV strains can be separated into 18 sub-species (Figure 3). The heterogeneity of CPXV was not only demonstrated between CPXV clusters, but it was also present within some clusters. Among them, CPXV-like 2 was the most heterogeneous. Their isolates were classified into ten sub-species based on the genetic and patristic distances. This clade comprised isolates of diverse geographic origins (Norway, Sweden, Denmark, UK, Germany and France) and its classification followed their geographical origin. Only German and French isolates were separated into more than one sub-species.

Large genetic variation was also found within VACV-like strains, which were closely related to VACV and MPXV, as previously described [38,40,41]. These strains split into three different sub-species based on the genetic and patristic distances. This division is in agreement with phylogenetic work reported in other previous studies [38,40,41]. Among VACV-like strains, it has been reported that CPXV-HumLit08/1 is a recombinant virus that contains genomic regions related to VACV, VACV-like and VARV-like [41]. However, our findings based on 62 non-recombinant conserved genes evidenced that CPXV-HumLit08/1 can be considered as one sub-species. Similarly, within ECTV-Abatino-like clade, CPXV-No-H2 has undergone recombination with other OPXV [40] and our data supported the separation of CPXV-No-H2 and the other ECTV-Abatino-like strains into different sub-species.

The most genetically homogeneous CPXV cluster was the VARV-like group. The origin of these strains was associated with infected pet rats, probably imported from the Czech Republic [37,82]. Overall, our findings are in concordance with the results of Mauldin et al. [38]. They reported that CPXV-like 1 strains were split into more than one cluster (referred in the study as E1, E2, E3, E4 and E5), VACV-like strains were divided into three groups (referred in the study as A, B and C) and VARV-like strains were clustered into a single group.

Despite the evidence of recombination in the datasets of 87 OPXV whole genomes, core genomes and orthologous genes, their phylogeny, genetic and patristic distances agreed with and are very similar to the phylogeny, patristic and genetic distances reconstructed from the dataset of 62 conserved genes without evidence of recombination. All four datasets suggested that CPXV strains can be divided into at least 18 sub-species (Figure 3, Figure S10 and Tables S6–S16). However, biological characterization of CPXV is required to accurately infer the taxonomic level to which these 18 sub-species of CPXV belong. Furthermore, our phylogenetic analysis evidenced that recombination did not change the phylogenetic relationship between CPXV strains and OPXV despite the extensive recombination between OPXV genomes. Taking into cognizance the extensive recombination present in CPXV genomes, it is rather surprising that recombination appears not to alter the clustering pattern in OPXV phylogeny. Plausible reasons may be that recombination among CPXVs occurred very early in CPXV/OPXV evolution, recombination regions occurred in small batch sizes compared to the whole genomes and the phylogenetic signals from recombinant regions were small and was diluted out by larger phylogenetic signals from other parts of the genome.

We estimated the evolution rate of CPXV based on 62 conserved genes of 55 CPXV to be 1.65 × 10^−5^ substitution/site/year (95% HPD, 4.36 × 10^−7^–4.32 × 10^−5^ subs/site/year). The 95% HPD of our estimate overlapped the reported substitution rates of *Chordopoxvirinae*, 0.5–8.8 ×10^−6^ substitutions/site/year, and OPXV, 1.7–6.5 × 10^−6^ substitutions/site/year [45,46,47,49]. The divergence times of CPXV could not be accurately estimated using 62 conserved genes of 55 CPXV genomes (Figure S9), even using conserved central region (F4L-A24L) of CPXV genomes (data not shown), since the broad 95% HPD intervals of the divergence time were quite broad. It could be due to the high heterogeneity of CXPV strains and the limited number of samples in terms of location, host and sample age. The majority of CPXV strains were isolated in Germany and from infections in humans. Furthermore, most CPXV strains were isolated in the last decades, there were no ancient CPXV isolates. Therefore, the low genetic information and the high genetic distances between the current CPXV strains increase the uncertainty of the node ages. In our opinion, our result strengthens the proposed idea that lineages of CPXV are highly divergent and a reclassification is needed, rather than showing a lack of a good calibration (tempest indicated presence of temporal signal). It has been proposed that the CXPV-like virus was the ancestor of Old World OPXV, excluding AKPV and AKMV, [39,44,45,83] due to its large genome, broadest host range and the presence of the most orthopoxviral genes [42,43,83,84]. Thus, despite the exclusion of other OPXV in our analysis due to the lack of temporal signal in the dataset, the evolutionary analysis of only CPXV may reflect the genomic history of all OPXV taking into account the high genetic heterogeneity among CPXV, the suggestion that CPXV or cowpox-like virus may be the ancestor to Old World OPXV species and the phylogenetic evidence of CPXV being the only OPXV that clusters with all Old World OPXV. However, the phylodynamic analysis of only CPXV has limitations because of oversampling of CPXV strains from Germany, from human zoonotic events and lack of ancient isolates. To improve the reconstruction of the evolutionary history of CPXV, increased genomic surveillance of CPXV across different regions of Eurasia and in multiple species or by the acquisition of ancient CPXV strains are required. These will result in a more accurate estimation of the time-scale of CPXV evolution.

## 5. Conclusions

In conclusion, the present study demonstrated the high genetic heterogeneity among CPXV isolates and the polyphyletic character of CPXV. Furthermore, our findings confirmed that CPXV was not a single species but a polyphyletic assemblage of several (up to 18) sub-species. Therefore, the current classification of CPXV as one single species should be re-evaluated. We also provided the first reconstruction of the evolutionary history of only CPXV. Overall, this study has shed significant insight on the evolution, phylogeny and classification of CPXV.

## Figures and Tables

**Table 1:** Genome size, number of predicted coding sequences (CDS) and genome coverage of the Fennoscandian CPXV isolates sequenced in this study.

Name	Genome Size (bp)	CDS	Genome Coverage	
			Illumina	Nanopore
CPXV-No-H1	221,926	215	300X	600X
CPXV-No-F1	221,334	217	820X	1519X
CPXV-No-F2	222,178	217	940X	1480X
CPXV-Swe-H1	220,981	217	700X	2500X
CPXV-Swe-H2	220,808	217	990X	2400X

## Data Availability

The original contributions presented in the study are publicly available. These data can be found here: https://www.ncbi.nlm.nih.gov/genbank/ (accessed on 2 August 2022), OP125537, OP125538, OP125539, OP125540, OP125541.

## Supplementary Materials

The following supporting information can be downloaded at: https://www.mdpi.com/article/10.3390/v14102134/s1, Figure S1. Presence of phylogenetic signal was evaluated by likelihood mapping checking for alternative topologies (tips), unresolved quartets (center) and partly resolved quartets (edges) for 87 OPXV whole genomes (a), core genomes (b), OPXV orthologous genes (c) and 62 conserved genes. Figure S2. Phylogenetic and temporal signal analyses. (a) Presence of phylogenetic signal was evaluated by likelihood mapping checking for alternative topologies (tips), unresolved quartets (center) and partly resolved quartets (edges) for 62 conserved genes of 55 CPXV strains. (b) Linear regression of root-to-tip genetic distance in a maximum likelihood phylogeny against sampling time for 62 conserved genes of 55 CPXV strains. Figure S3. Recombination analysis of 87 orthopoxvirus (OPXV) core genomes with RPD4. Schematic sequence display depicting color-coded representations of the analyzed sequences and the locations of detected recombination events in the 87 OPXV core genomes. The detected recombination events were detected for at least 5 of 7 methods (RDP, GENECONV, Bootscan, MaxChi, Chimaera, SiScan and 3Seq) with significant *p*-values (*p* ≤ 0.01). Figure S4. Bayesian inference phylogenetic tree of 62 conserved genes from 87 orthopoxviruses. Diamonds at the nodes indicate posterior probabilities > 0.9. The scale bar represents expected substitutions per site. The main five cowpox virus (CPXV) clusters were highlighted in different colors: pink (Ectromelia-Abatino-like CPXV), blue (CPXV-like 1), green (CPXV-like 2), turquoise blue (Vaccinia-like CPXV) and orange (Variola-like CPXV). Figure S5. Bayesian inference phylogenetic tree of 87 OPXV core genomes. Posterior probabilities are shown on the right side of each node and only posterior probabilities above 0.9 are shown. The scale bar represents expected substitutions per site. The main five cowpox virus (CPXV) clusters were highlighted in different colors: pink (Ectromelia-Abatino-like CPXV), blue (CPXV-like 1), green (CPXV-like 2), turquoise blue (Vaccinia-like CPXV) and orange (Variola-like CPXV). Figure S6. Maximum likelihood phylogenetic tree of 87 orthopoxvirus whole genome. Bootstrap values were inferred from 1000 rapid bootstrap replicates. Diamonds at the nodes indicate bootstrap values > 80%. The scale bar indicates substitution per site. The main five cowpox virus (CPXV) clusters were highlighted in different colors: pink (Ectromelia-Abatino-like CPXV), blue (CPXV-like 1), green (CPXV-like 2), turquoise blue (Vaccinia-like CPXV) and orange (Variola-like CPXV). Figure S7. Bayesian inference phylogenetic tree of 87 OPXV whole genomes. Diamonds at the nodes indicate posterior probabilities > 0.9. The scale bar represents expected substitutions per site. The main five cowpox virus (CPXV) clusters were highlighted in different colors: pink (Ectromelia-Abatino-like CPXV), blue (CPXV-like 1), green (CPXV-like 2), turquoise blue (Vaccinia-like CPXV) and orange (Variola-like CPXV). Figure S8. Maximum likelihood phylogenetic tree based on orthopoxvirus orthologous genes. Bootstrap values were inferred from 1000 rapid bootstrap replicates. Diamonds at the nodes indicate bootstrap values > 80%. The scale indicates substitution per site. The main five cowpox virus (CPXV) clusters were highlighted in different colors: pink (Ectromelia-Abatino-like CPXV), blue (CPXV-like 1), green (CPXV-like 2), turquoise blue (Vaccinia-like CPXV) and orange (Variola-like CPXV). Figure S9. Bayesian maximum clade credibility (MCC) tree of 62 non-recombinant conserved genes of 55 CPXV genomes. The MCC tree was generated using BEAST 1, using a log-normal relaxed clock, coalescent Bayesian skyline population, HKY substitution model and four gamma categories. The numbers on the nodes indicate the time of the most recent common ancestor of the clades. Diamonds at the nodes indicate posterior probability values > 0.9. The main five CPXV clusters were highlighted in different colors: pink (Ectromelia-Abatino-like CPXV), blue (CPXV-like 1), green (CPXV-like 2), turquoise blue (Vaccinia-like CPXV) and orange (Variola-like CPXV). Figure S10. New classification of Cowpox virus (CPXV) based on phylogenetic inference (from 87 OPXV whole genomes, core genomes and orthologous genes), patristic and genetic distances. Diamonds at the nodes indicate bootstrap values > 80%. The main five CPXV clusters were highlighted in different colors: pink (Ectromelia-Abatino-like CPXV), blue (CPXV-like 1), green (CPXV-like 2), turquoise blue (Vaccinia-like CPXV) and orange (Variola-like CPXV). Table S1: List of strains used in the phylogenetic analysis. Table S2: List of orthologous genes from 87 orthopoxviruses used in this study. Table S3: List of 62 conserved genes from 87 orthopoxviruses used in this study. Table S4: List of strains used in the evolution molecular analysis. Table S5. Predicted genes in CPXV-No-F1, CPXV-No-F2, CPXV-No-H1, CPXV-Swe-H1 and CPXV-Swe-H2 compared to reference genomes CPXV-Brighton (CPXV_BR). Table S6. Patristic distances between CPXV clusters and OPXV species calculated from the Maximum likelihood (ML) and Bayesian inference (BI) trees of 62 conserved genes, 87 OPXV whole genomes, core genomes and orthologous genes. Table S7. Genetic distances between CPXV clusters and OPXV species estimated by p-distances from the alignment of 62 conserved genes (A), 87 OPXV whole genomes (B), core genomes (C) and orthologous genes (D). Table S8. Patristic and genetic distances within CPXV clusters calculated from the Maximum likelihood (ML) and Bayesian inference (BI) trees of 62 conserved genes, 87 OPXV core genomes, whole genomes and orthologous genes and their alignments, respectively. Table S9. Patristic distances within CPXV-like 2 calculated from the Maximum likelihood (ML) and Bayesian inference (BI) trees of 62 conserved genes, 87 OPXV whole genomes, core genomes and orthologous genes. Table S10. Genetic distances within CPXV-like 2 estimated by p-distances from the alignment of 62 conserved genes (A), 87 OPXV whole genomes (B), core genomes (C) and orthologous genes (D). Table S11. Patristic and genetic distances within ECTV-Abatino-like calculated from the Maximum likelihood (ML) and Bayesian inference (BI) trees of 62 conserved genes, 87 OPXV whole genomes, core genomes and orthologous genes and their alignments, respectively. Table S12. Patristic distances within VACV-like calculated from the Maximum likelihood (ML) and Bayesian inference (BI) trees of 62 conserved genes, 87 OPXV whole genomes, core genomes and orthologous genes. Table S13. Genetic distances within VACV-like clade estimated by p-distances from the alignment of 62 conserved genes (A), 87 OPXV whole genomes (B), core genomes (C) and orthologous genes (D). Table S14. Patristic and genetic distances within CPXV-like 1 calculated from the Maximum likelihood (ML) and Bayesian inference (BI) trees of 87 OPXV whole genomes, core genomes and orthologous genes and their alignments, respectively. Table S15. Patristic distances within CPXV-like 1 calculated from the Maximum likelihood (ML) and Bayesian inference (BI) trees of 62 conserved genes, 87 OPXV whole genomes, core genomes and orthologous genes. Table S16. Genetic distances within CPXV-like 1 estimated by p-distances from the alignment of 62 conserved genes (A), 87 OPXV whole genomes (B), core genomes (C) and orthologous genes (D).
