# Supplementary material for: Genomic Sequencing and Phylogenomics of Cowpox Virus
Source: Viruses. 2022 Sep 28;14(10):2134. doi: 10.3390/v14102134 (PMC9611595; doi:10.3390/v14102134)
Supplement: Supplementary file 1 [file viruses-14-02134-s001.zip › Final_Revised_Supplementary_information.pdf]

Supplementary information

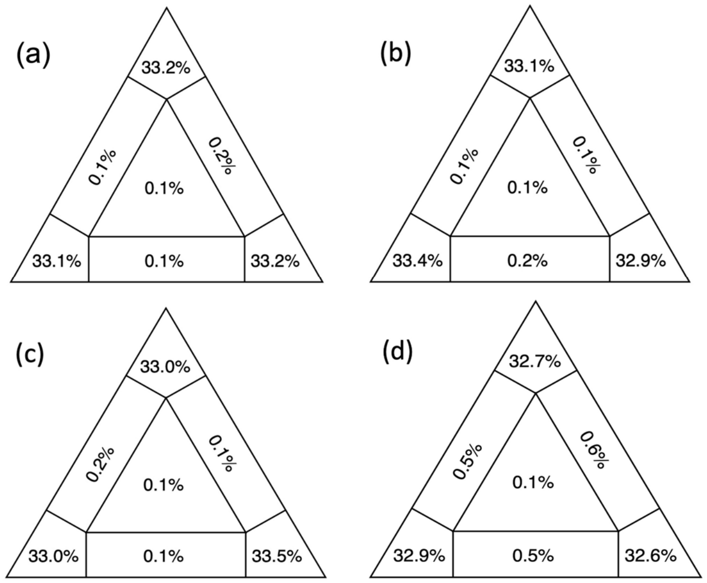

Figure S1. Presence of phylogenetic signal was evaluated by likelihood mapping checking for alternative topologies (tips), unresolved quartets (center) and partly resolved quartets (edges) for 87 OPXV whole genome (a), core genome (b), OPXV orthologous genes (c) and 62 conserved genes.

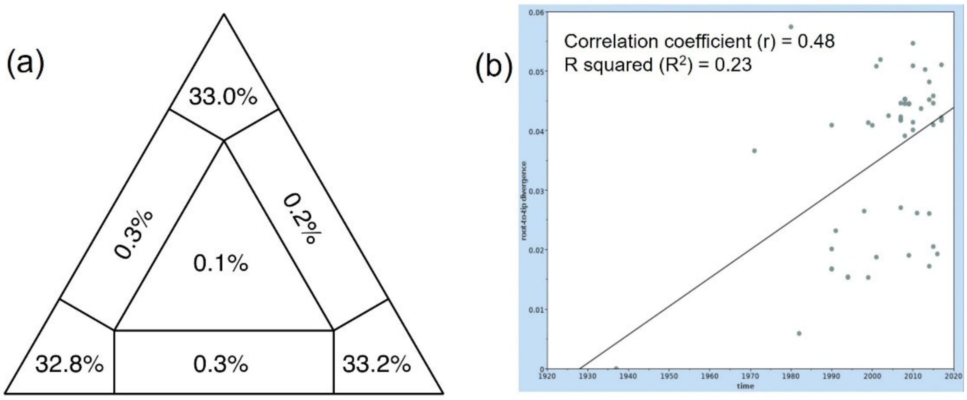

Figure S2. Phylogenetic and temporal signal analyses. (a) Presence of phylogenetic signal was evaluated by likelihood mapping checking for alternative topologies (tips), unresolved quartets (centre) and partly resolved quartets (edges) for 62 conserved genes of 55 CPXV strains. (b) Linear regression of root-to-tip genetic distance in a maximum likelihood phylogeny against sampling time for 62 conserved genes of 55 CPXV strains.

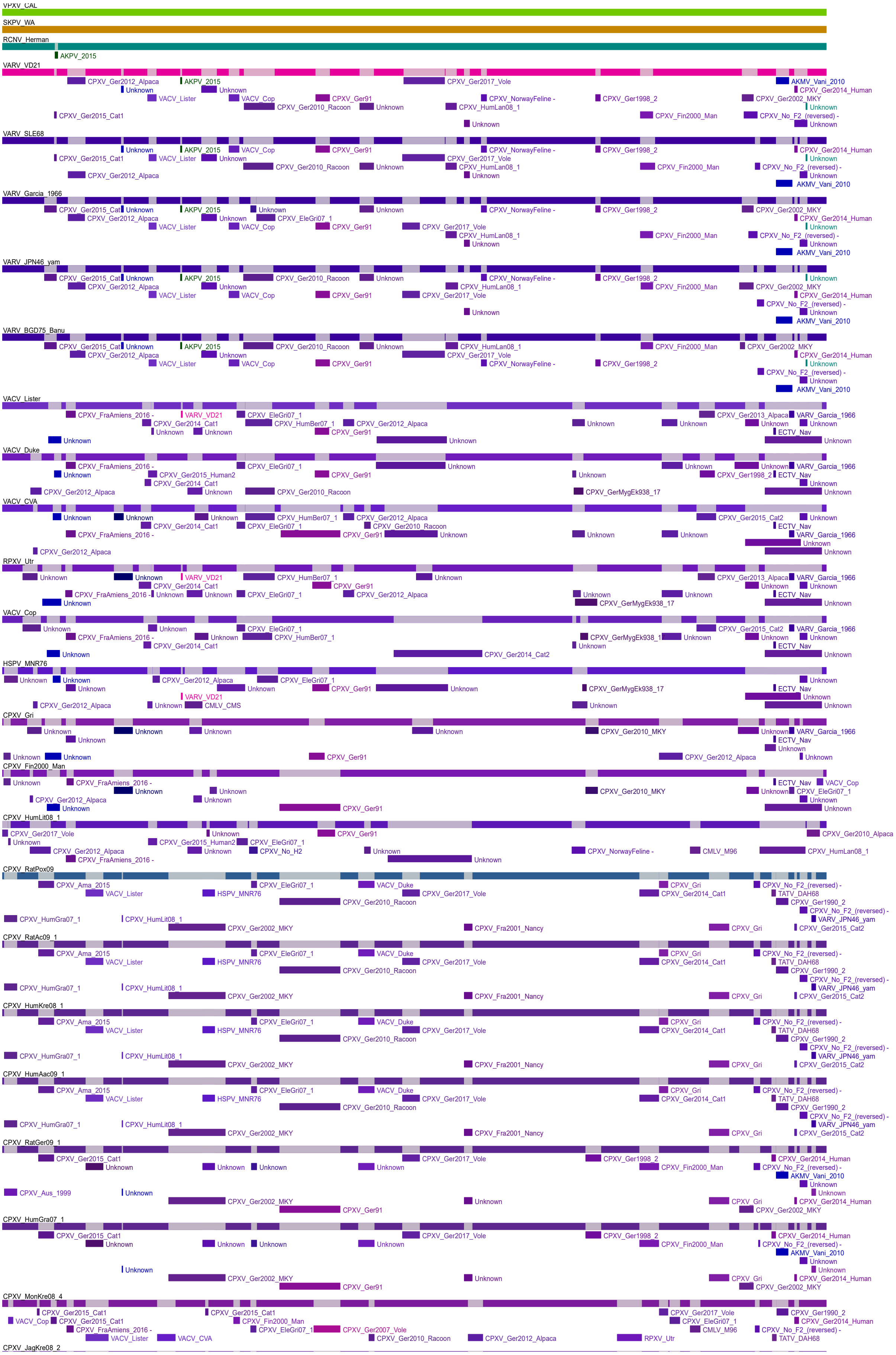
VPXV_CAL
SKPV_WA
RCNV_Herman
VARV_VD21
VARV_SLE68
VARV_Garcia_1966
VARV_JPN46_yam
VARV_BGD75_Banu
VACV_Lister
VACV_Duke
VACV_CVA
RPXV_Utr
VACV_Cop
HSPV_MNR76
CPXV_Gri
CPXV_Fin2000_Man
CPXV_HumLit08_1
CPXV_RatPox09
CPXV_RatAc09_1
CPXV_HumKre08_1
CPXV_HumAac09_1
CPXV_RatGer09_1
CPXV_HumGra07_1
CPXV_MonKre08_4
CPXV_JagKre08_2

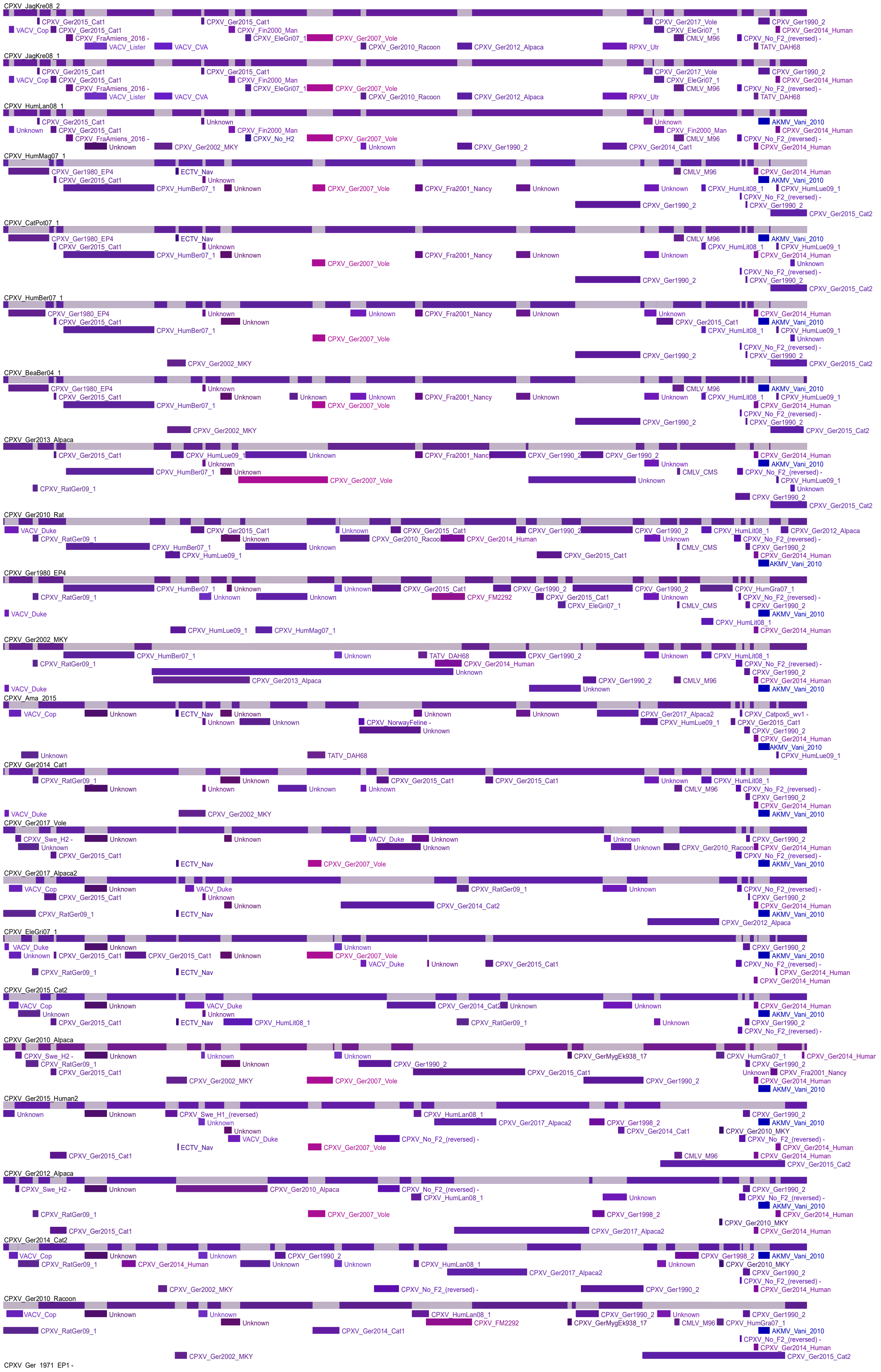

CPXV_JagKre08_2
CPXV_Ger2015_Cat1
CPXV_Ger2015_Cat1
CPXV_Ger2017_Vole
CPXV_Ger1990_2
VACV_Cop
CPXV_Ger2015_Cat1
CPXV_Fin2000_Man
CPXV_EleGri07_1
CPXV_Ger2014_Human
CPXV_FraAmiens_2016 -
CPXV_EleGri07_1
CPXV_Ger2007_Vole
CMLV_M96
CPXV_No_F2_(reversed) -
VACV_Lister
VACV_CVA
CPXV_Ger2010_Racoon
CPXV_Ger2012_Alpaca
RPXV_Utr
TATV_DAH68
CPXV_JagKre08_1
CPXV_HumLan08_1
Unknown
AKMV_Vani_2010
CPXV_No_H2
CPXV_Ger2002_MKY
CPXV_Ger1990_2
CPXV_Ger2014_Cat1
CPXV_HumMag07_1
CPXV_Ger1980_EP4
ECTV_Nav
CPXV_HumBer07_1
CPXV_Fra2001_Nancy
CPXV_HumLit08_1
CPXV_HumLue09_1
CPXV_Ger2015_Cat2
CPXV_CatPot07_1
CPXV_HumBer07_1
CPXV_BeaBer04_1
CPXV_Ger2013_Alpaca
CMLV_CMS
CPXV_RatGer09_1
CPXV_Ger2010_Rat
VACV_Duke
CPXV_Ger2010_Racoo
CPXV_Ger1980_EP4
CPXV_FM2292
CPXV_HumGra07_1
CPXV_HumMag07_1
CPXV_Ger2002_MKY
TATV_DAH68
CPXV_Ama_2015
CPXV_NorwayFeline -
CPXV_Ger2017_Alpaca2
CPXV_Catpox5_wv1 -
CPXV_Ger2014_Cat1
CPXV_Ger2017_Vole
CPXV_Swe_H2 -
CPXV_Ger2017_Alpaca2
CPXV_Ger2014_Cat2
CPXV_EleGri07_1
CPXV_Ger2015_Cat2
CPXV_Ger2010_Alpaca
CPXV_GerMygEk938_17
CPXV_Ger2015_Human2
CPXV_Swe_H1_(reversed)
CPXV_Ger1998_2
CPXV_Ger2010_MKY
CPXV_Ger2012_Alpaca
CPXV_Ger2010_Racoon
CPXV_Ger_1971_EP1 -

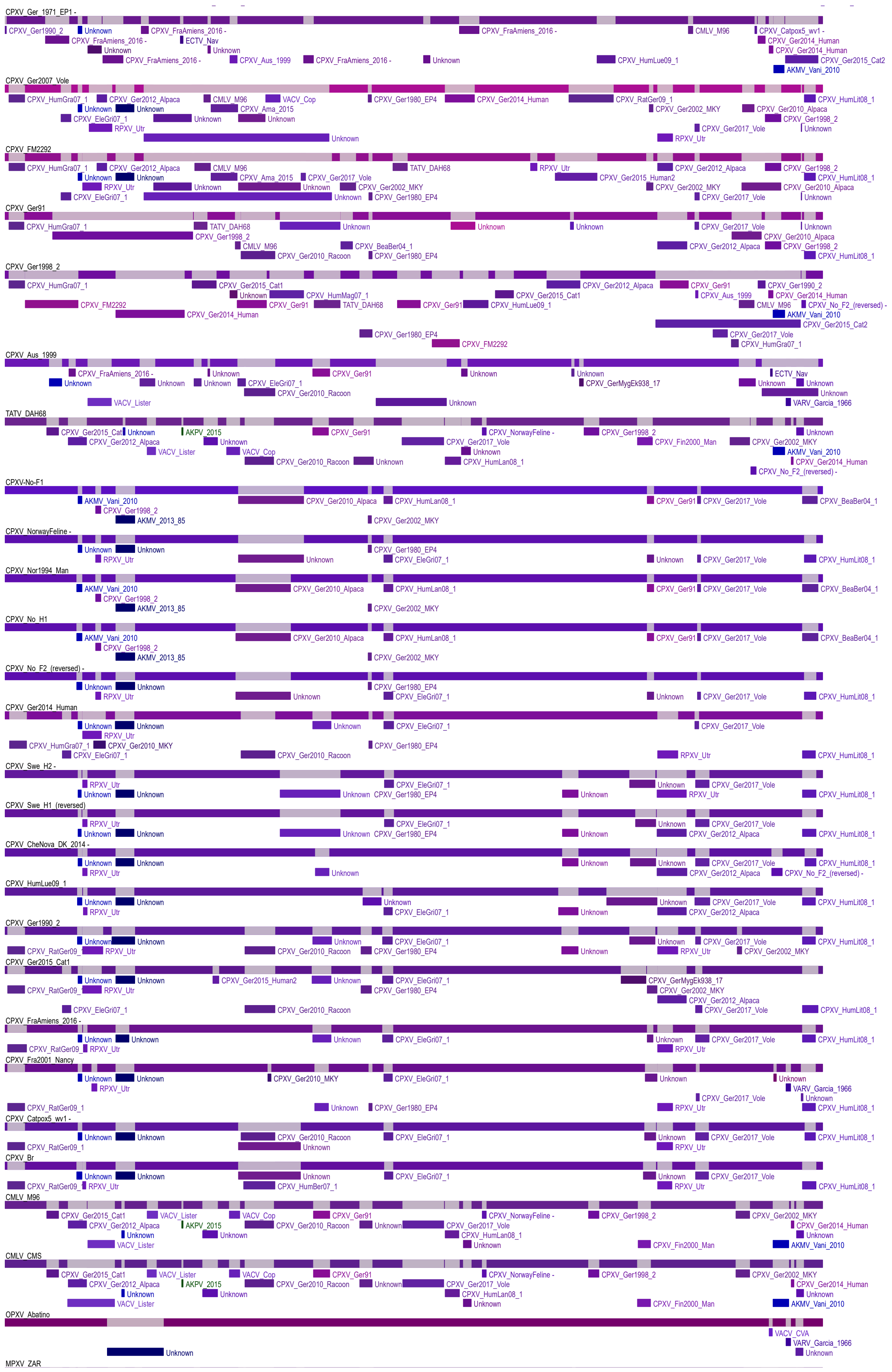
CPXV_Ger_1971_EP1 -
CPXV_Ger2007_Vole
CPXV_FM2292
CPXV_Ger91
CPXV_Ger1998_2
CPXV_Aus_1999
TATV_DAH68
CPXV-No-F1
CPXV_NorwayFeline -
CPXV_Nor1994_Man
CPXV_No_H1
CPXV_No_F2_(reversed) -
CPXV_Ger2014_Human
CPXV_Swe_H2 -
CPXV_Swe_H1_(reversed)
CPXV_CheNova_DK_2014 -
CPXV_HumLue09_1
CPXV_Ger1990_2
CPXV_Ger2015_Cat1
CPXV_FraAmiens_2016 -
CPXV_Fra2001_Nancy
CPXV_Catpox5_wv1 -
CPXV_Br
CMLV_M96
CMLV_CMS
OPXV_Abatino
MPXV_ZAR

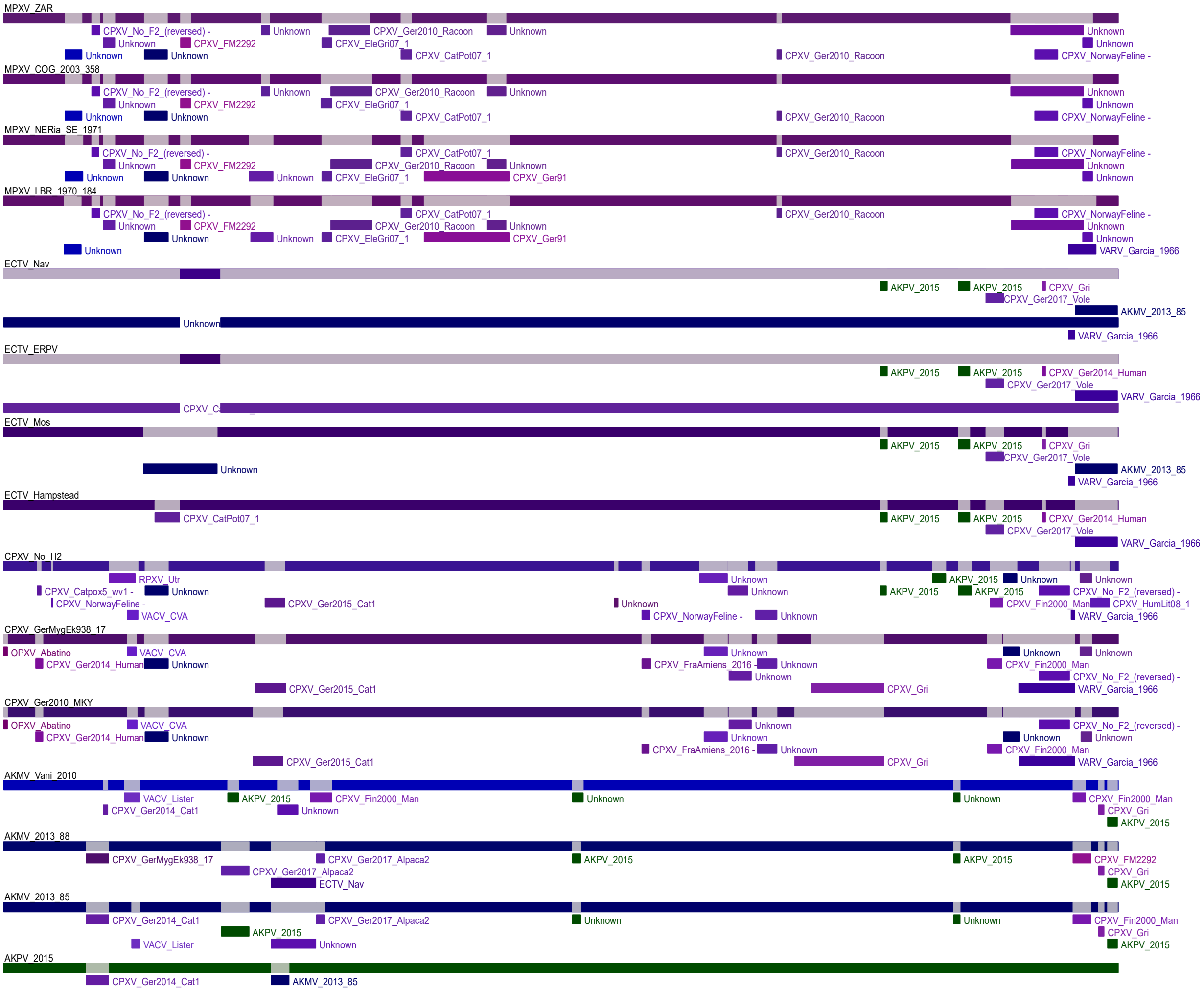

Figure S3. Recombination analysis of 87 orthopoxvirus (OPXV) core genomes with RPD4. Schematic sequence display depicting colour-coded representations of the analyzed sequences and the locations of detected recombination events in the 87 OPXV core genomes. The detected recombination events were detected for at least 5 of 7 methods (RDP, GENECONV, Bootscan, MaxChi, Chimaera, SiScan, and 3Seq) with significant p-values ($p \leq 0.01$).

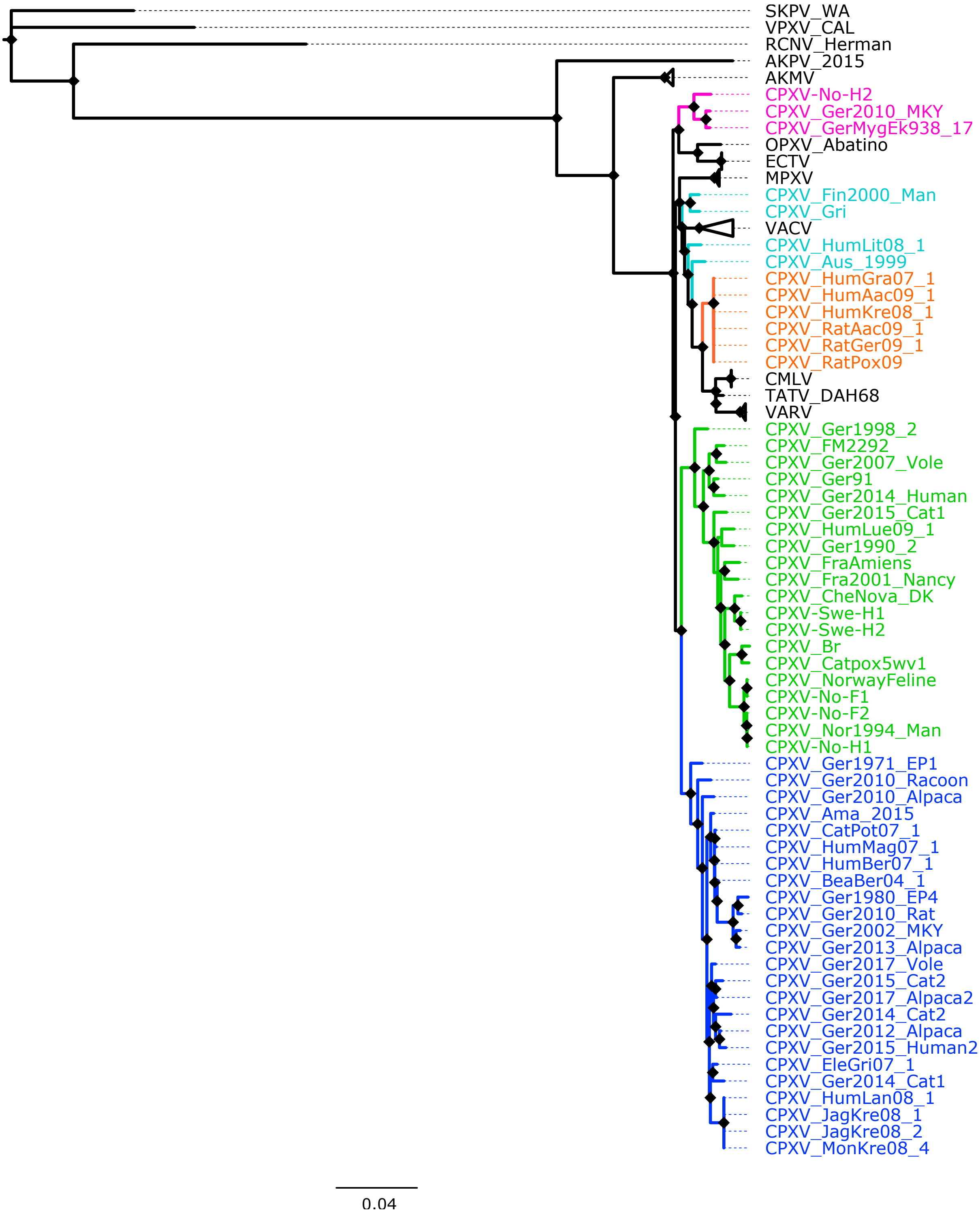

Figure S4. Bayesian inference phylogenetic tree of 62 conserved genes from 87 orthopoxviruses. Diamonds at the nodes indicate posterior probabilities >0.9. The scale bar represents expected substitutions per site. The main five cowpox virus (CPXV) clusters were highlighted in different colors: pink (Ectromelia-Abatino-like CPXV), blue (CPXV-like 1), green (CPXV-like 2), turquoise blue (Vaccinia-like CPXV) and orange (Variola-like CPXV).

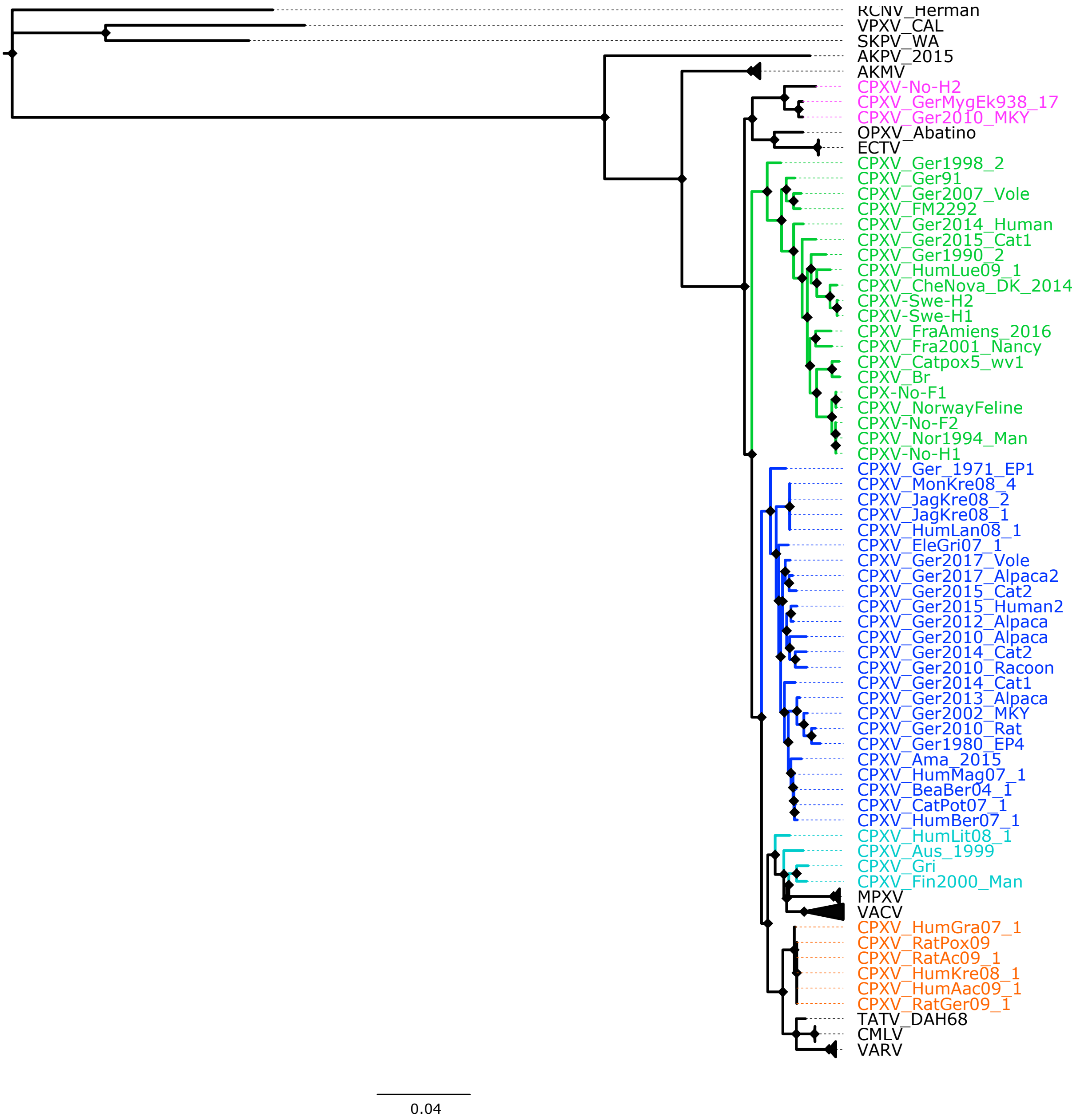

Figure S5. Bayesian inference phylogenetic tree of 87 OPXV core genomes. Posterior probabilities are shown on the right side of each node and only posterior probabilities above 0.9. are shown. The scale bar represents expected substitutions per site. The main five cowpox virus (CPXV) clusters were highlighted in different colors: pink (Ectromelia-Abatino-like CPXV), blue (CPXV-like 1), green (CPXV-like 2), turquoise blue (Vaccinia-like CPXV) and orange (Variola-like CPXV).

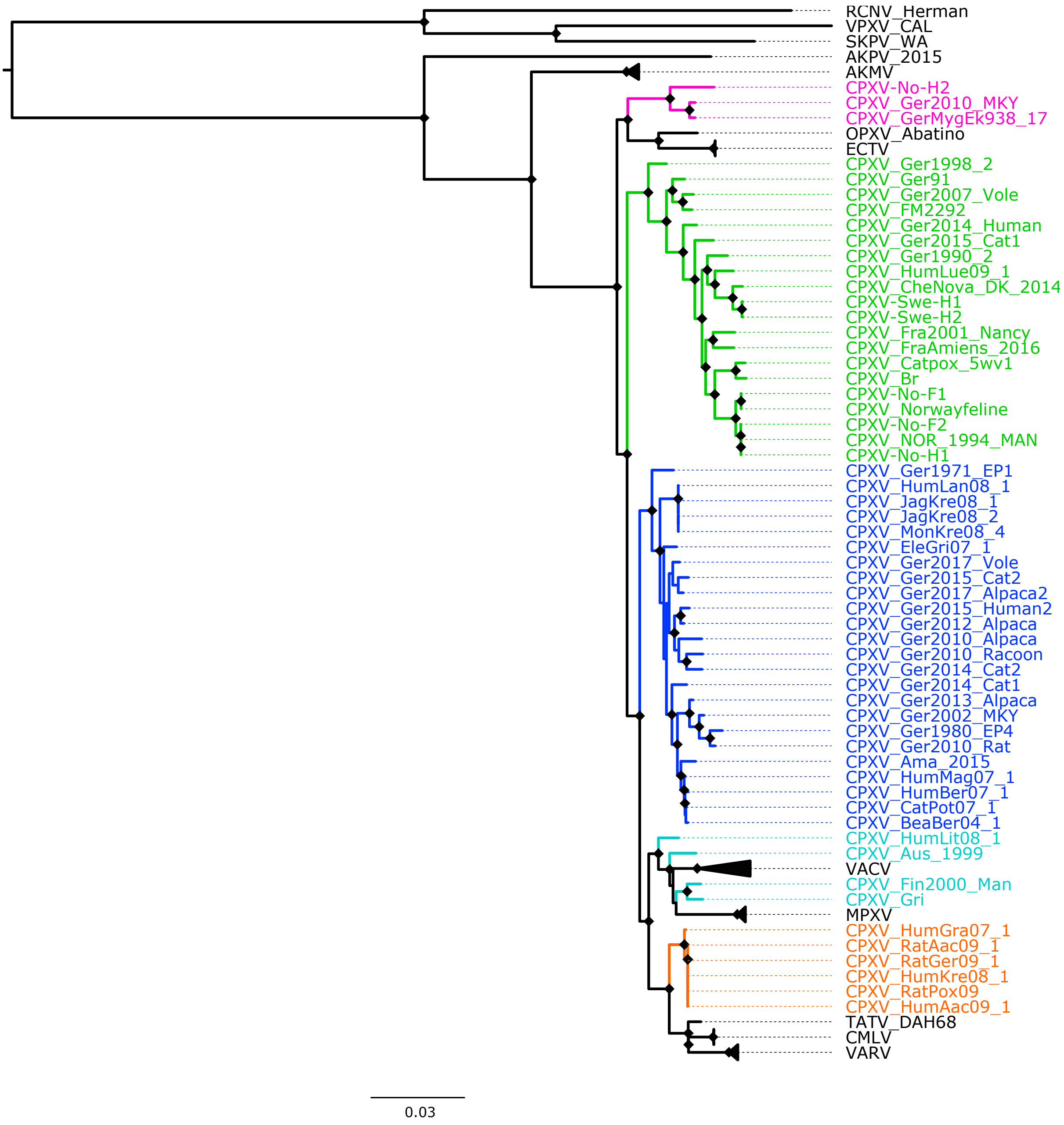

Figure S6. Maximum Likelihood phylogenetic tree of 87 orthopoxvirus whole genomes. Bootstrap values were inferred from 1000 rapid bootstrap replicates. Diamonds at the nodes indicate bootstrap values >80%. The scale bar indicates substitution per site. The main five cowpox virus (CPXV) clusters were highlighted in different colors: pink (Ectromelia-Abatino-like CPXV), blue (CPXV-like 1), green (CPXV-like 2), turquoise blue (Vaccinia-like CPXV) and orange (Variola-like CPXV).

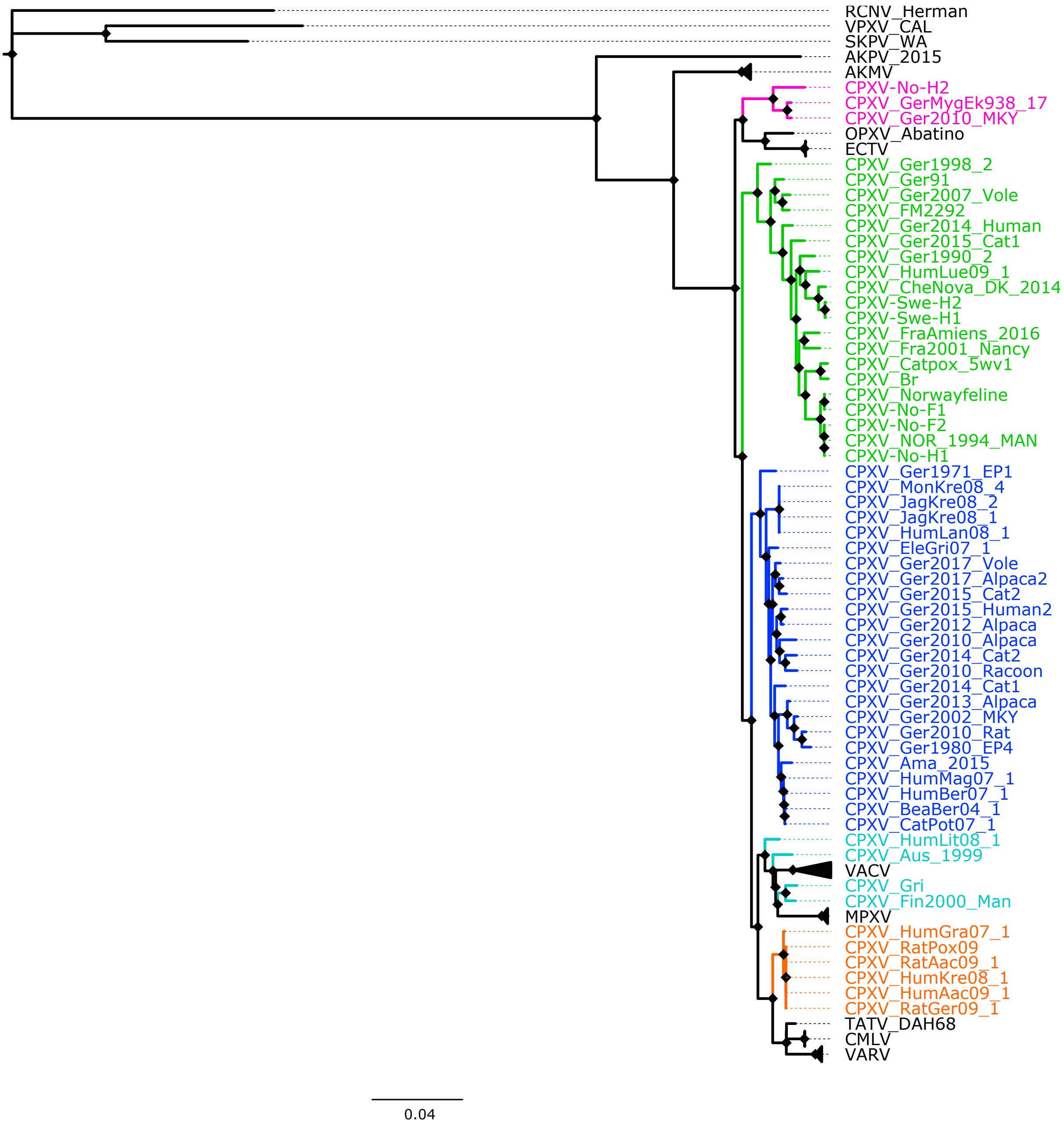

Figure S7. Bayesian inference phylogenetic tree of 87 OPXV whole genomes. Diamonds at the nodes indicate posterior probabilities > 0.9. The scale bar represents expected substitutions per site. The main five cowpox virus (CPXV) clusters were highlighted in different colors: pink (Ectromelia-Abatino-like CPXV), blue (CPXV-like 1), green (CPXV-like 2), turquoise blue (Vaccinia-like CPXV) and orange (Variola-like CPXV).

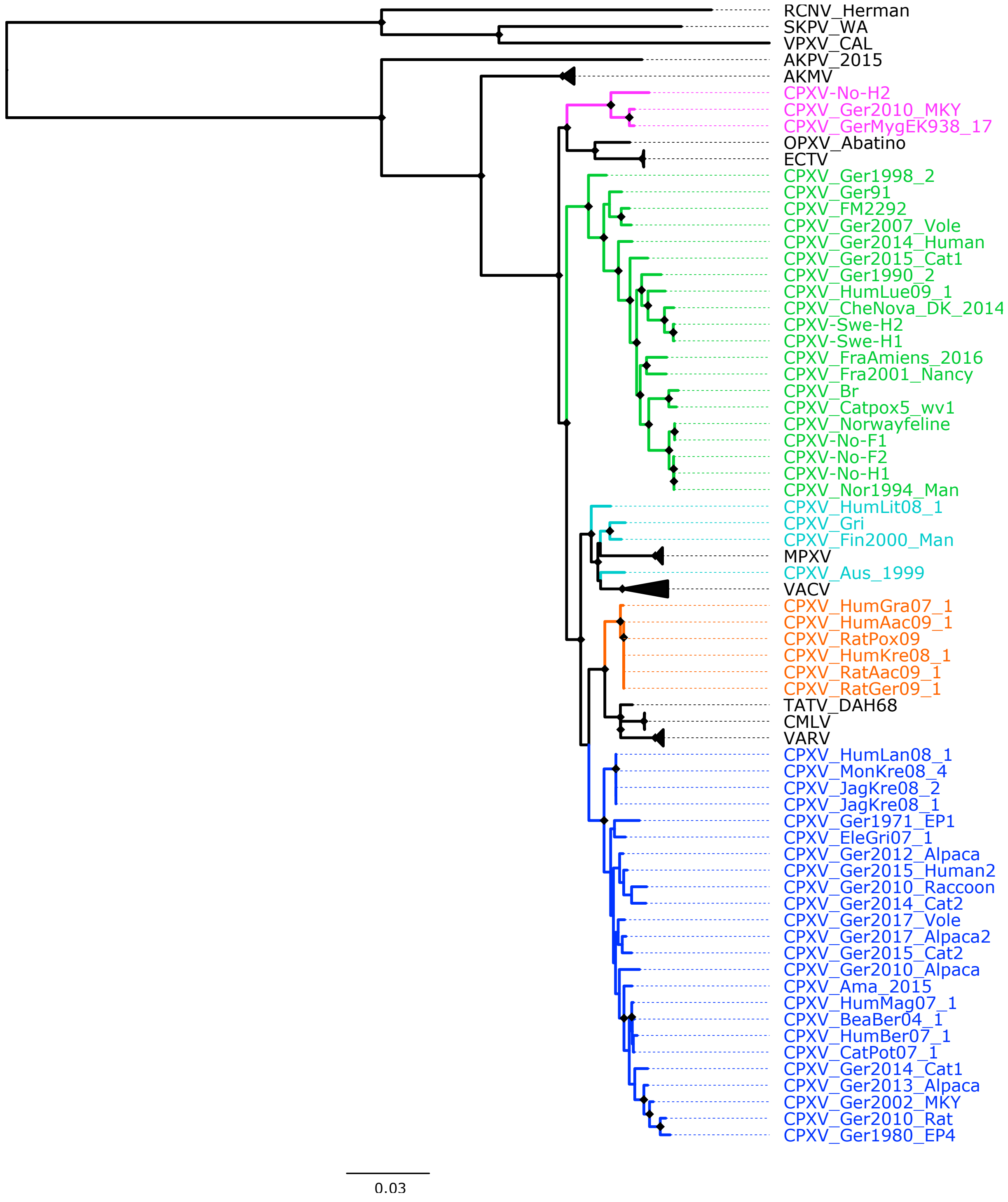

Figure S8. Maximum Likelihood phylogenetic tree based on orthopoxvirus orthologous genes. Bootstrap values were inferred from 1000 rapid bootstrap replicates. Diamonds at the nodes indicate bootstrap values >80%. The scale indicates substitution per site. The main five cowpox virus (CPXV) clusters were highlighted in different colors: pink (Ectromelia-Abatino-like CPXV), blue (CPXV-like 1), green (CPXV-like 2), turquoise blue (Vaccinia-like CPXV) and orange (Variola-like CPXV).

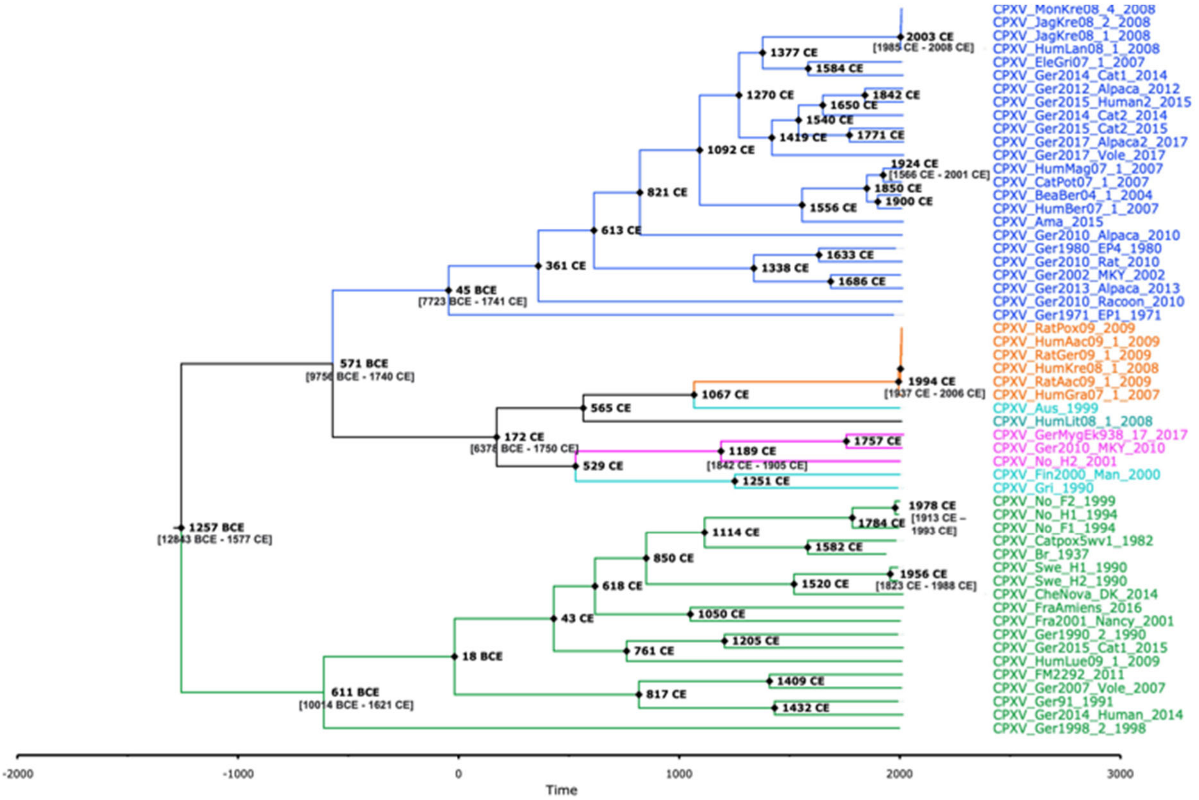

Figure S9. Bayesian maximum clade credibility (MCC) tree of 62 non-recombinant conserved genes of 55 CPXV genomes. The MCC tree was generated using BEAST 1, using a log-normal relaxed clock, coalescent Bayesian skyline population, HKY substitution model and four gamma categories. The numbers on the nodes indicate the time of the most recent common ancestor of the clades. Diamonds at the nodes indicate posterior probability values >0.9. The main five CPXV clusters were highlighted in different colors: pink (Ectromelia-Abatino-like CPXV), blue (CPXV-like 1), green (CPXV-like 2), turquoise blue (Vaccinia-like CPXV) and orange (Variola-like CPXV).

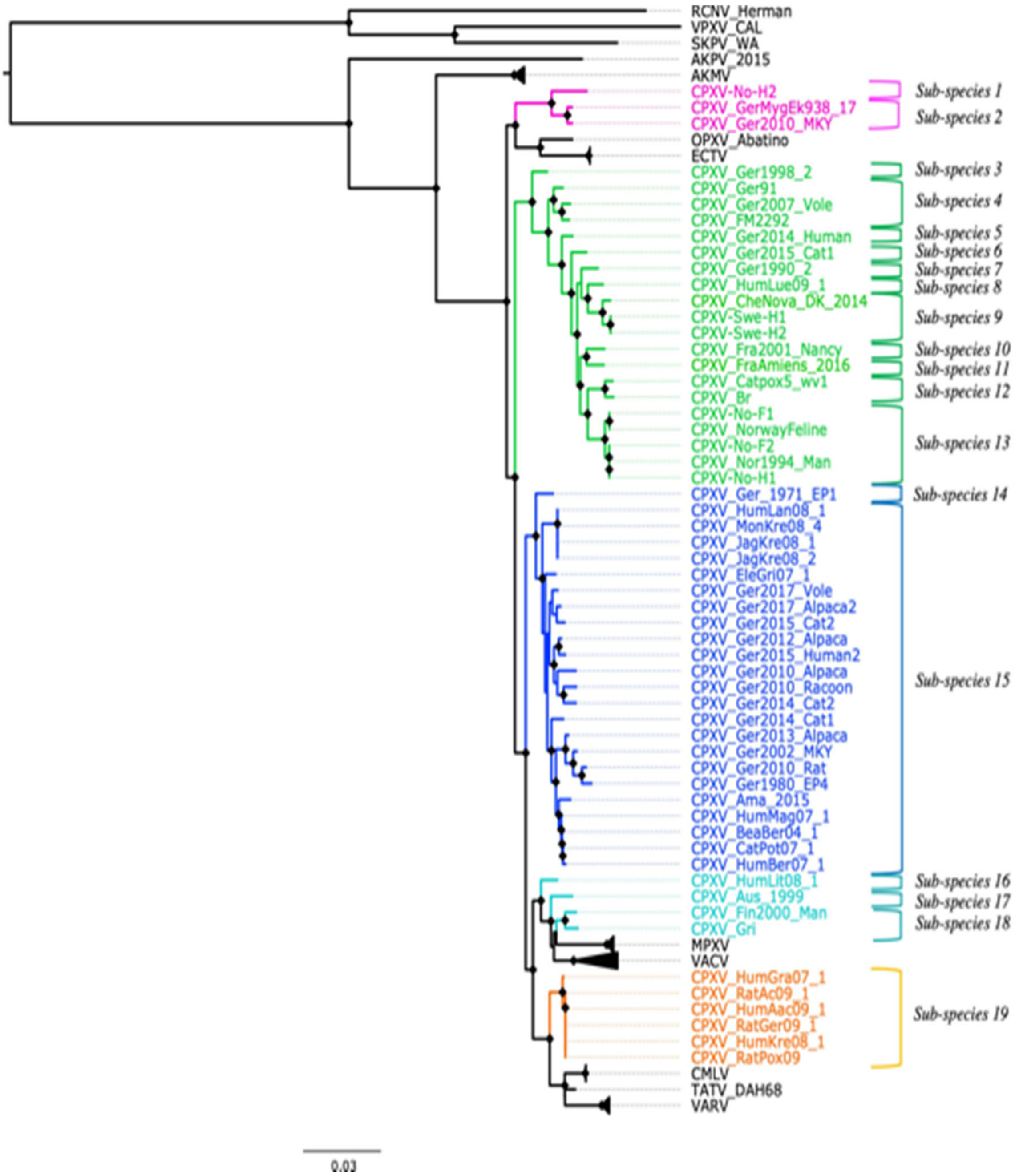

Figure S10. New classification of cowpox virus (CPXV) based on phylogenetic inference (from 87 OPXV whole genomes, core genomes and orthologous genes), patristic and genetic distances. Diamonds at the nodes indicate bootstrap values >80%. The main five CPXV clusters were highlighted in different colors: pink (Ectromelia-Abatino-like CPXV), blue (CPXV-like 1), green (CPXV-like 2), turquoise blue (Vaccinia-like CPXV) and orange (Variola-like CPXV).
